# Structural basis of collagen glucosyltransferase function and its serendipitous role in kojibiose synthesis

**DOI:** 10.21203/rs.3.rs-5850681/v1

**Published:** 2025-01-29

**Authors:** Jeong Seon Kim, Zhenhang Chen, Sara Andrea Espinosa Garcia, Christoph Buhlheller, Stephen J. Richards, Tingfei Chen, Jingjing Wu, Ronald C. Bruntz, Marisa E. Gilliam, Mitsuo Yamauchi, Bo Liang, Houfu Guo

**Affiliations:** 1 Department of Molecular and Cellular Biochemistry, University of Kentucky, Lexington, KY, USA; Markey Cancer Center, University of Kentucky, Lexington, KY, USA.; 2 Department of Biochemistry, Emory University School of Medicine, Atlanta, GA, USA.; 3 Medical University of Graz, Graz, Austria.; 4 Department of Biomedical Sciences, Adams School of Dentistry, University of North Carolina at Chapel Hill, Chapel Hill, NC, USA.

**Keywords:** lysyl hydroxylase, glycosylation, lysyl post-translational modifications, DNA viruses

## Abstract

Collagen glucosyltransferases catalyze a unique type of collagen glucosylation that is critical for biological processes and disease mechanisms. However, the structural regulation of collagen glucosyltransferases remains poorly understood. Here, we report the crystal structures of a mimiviral collagen glucosyltransferase in its apo form and in complexes with uridine diphosphate (UDP) and the disaccharide product. Our findings reveal that the enzyme functions as a homodimer, stabilized by a loop from one subunit locking into a cleft on the opposite subunit. This dimerization enables UDP-glucose binding cooperativity and enzymatic activity, a property conserved in the human homolog. Further structural analyses suggest an induced fit model for UDP interaction, mediated by Lysine 222. The dimerization also forms an extended cleft flanked by two active sites, which likely facilitates collagen recognition. Unexpectedly, we discovered that the mimiviral collagen glucosyltransferase can also synthesize the prebiotic disaccharide kojibiose. An elongated pocket adjacent to the UDP-binding site allows the enzyme to use UDP-glucose as the sugar donor and glucose as the acceptor for kojibiose production. Enzymatic activity assays confirmed the enzyme’s novel kojibiose synthesis activity *in vitro* and *in vivo*. These structural insights not only inform glucosyltransferase function but also open new avenues for biomedicine.

## Introduction

Collagens are the most abundant protein family in vertebrates by mass, performing a wide range of structural and biological functions (1). During their biosynthesis, collagens acquire a series of lysine (Lys) post-translational modifications that are crucial for their functions (2,3). Specific collagen lysine residues can be hydroxylated by lysyl hydroxylases, which are encoded by the *procollagen-lysine 2-oxoglutarate 5-dioxygenase* genes (*PLODs)*, to form 5-hydroxylysine (Hyl). Certain Hyl residues in the collagen helical domain are further galactosylated by glycosyltransferase 25 domain-containing proteins 1 and 2 (GLT25D1 and GLT25D2) (4). These galactosylated Hyl residues can be further glucosylated to form the unique Hyl-O-linked disaccharide, 2-O-α-D-galactopyranosyl-D-glucose (also known as 4′-epi-kojibiose) (5). Collagen glucosylation is catalyzed by galactosylhydroxylysyl glucosyltransferases (GGTs), which are encoded by the same polypeptides as lysyl hydroxylases (6–9). This glucosylation process plays key roles in regulating fibrillogenesis, matrix mineralization, axon guidance, angiogenesis, platelet aggregation, and metastasis (6,9–13). Dysregulation of Lys modifications is associated with various diseases. For example, mutations in the *PLOD2* gene cause Bruck syndrome II, a rare form of osteogenesis imperfecta characterized by joint contractures (14). Conversely, increased *PLOD2* expression has been linked to fibrosis and cancer progression (2,15).

The levels of collagen lysyl post-translational modifications are tightly regulated and play critical roles in both normal biology and disease. Studies in human patients with connective tissue disorders and heterozygous *PLOD3* knock-out mice have shown that even a moderate reduction in *PLOD3* levels leads to connective tissue abnormalities accompanied by developmental defects (16–19). Our recent work has uncovered collagen glucosyltransferase (GGT) activity in proteins encoded by *PLOD1* and an isoform of *PLOD2* known as *PLOD2b*, which arises from alternative splicing of *PLOD2* pre-mRNA (9). The GGT activity of *PLOD1* protein has been linked to vascular development (20). In contrast, *PLOD2b* protein, characterized by the inclusion of exon 13a, exhibits cooperative binding of its co-substrate, uridine diphosphate glucose (UDP-glucose), with a higher affinity and demonstrates higher collagen glucosyltransferase activity compared to *PLOD2a* protein, the isoform lacking exon 13a (9). *PLOD2b* is specifically upregulated in fibroblasts—the primary collagen-producing cells—during mesenchymal differentiation. This isoform is associated with epithelial-to-mesenchymal transition (EMT) and plays a critical role in promoting cancer progression of multiple types (9,21–25). Since UDP-glucose levels are often depleted during mesenchymal differentiation, the cooperative binding of UDP-glucose by *PLOD2b* protein may enable efficient glucosylation of fibrillar collagen in fibroblasts under these conditions. However, the structural basis of collagen glucosyltransferase’s substrate binding and cooperativity remains largely unknown.

Due to their critical roles in tissue homeostasis, collagen and collagen lysyl modifying enzymes are highly conserved in the animal kingdom, from humans to sponges (5,26). Remarkably, collagen-like proteins and collagen-modifying enzymes have also been identified in certain fungi, bacteria, and viruses, including *Acanthamoeba polyphaga* mimivirus (5,27). Structural and functional studies of mimiviral collagen lysyl hydroxylase have provided valuable insights into the functions of human collagen-modifying enzymes (27,28). Our previous work demonstrated that the active domain of mimiviral lysyl hydroxylase forms a dimer assembly similar to that of human *PLOD* family members (28). More recently, genomic and biochemical analyses have identified a mimiviral collagen glucosyltransferase, R699, capable of modifying galactosyl Hyl residues in collagen (29). These findings suggest that collagens and their lysyl post-translational modifications are conserved beyond the animal kingdom. Interestingly, during the life cycle of amoeba, the natural host of mimivirus, the levels of UDP-glucose fluctuate significantly (30). However, it remains unclear whether mimivirus possesses mechanisms to sense and respond to these changes in UDP-glucose levels.

In this study, we present a series of high-resolution crystal structures of mimiviral collagen glucosyltransferase, complemented by protein biochemical studies. These structures, with and without UDP and the disaccharide product, provide key snapshots of critical events in the enzyme’s function. Our structural analyses revealed that mimiviral collagen glucosyltransferase forms a dimer, which binds UDP-glucose cooperatively. Comparisons between structures with and without UDP highlight changes in active site electrostatics, supporting an induced-fit model for UDP-glucose binding. Additionally, the dimer appears to form a continuous collagen-binding site, potentially enabling the enzyme to recognize and process long collagen peptides. These structural features are conserved in collagen glucosyltransferases derived from animals, underscoring their functional significance. Unexpectedly, we also discovered a novel kojibiose synthase activity in the mimiviral collagen glucosyltransferase, demonstrated both *in vitro* and *in vivo*. Structural analysis revealed that the disaccharide product, kojibiose, is engaged by an elongated sugar-binding pocket adjacent to the UDP-binding site. Together, these findings provide valuable structural insights into the functions of collagen glucosyltransferases and open new intriguing avenues for understanding their roles in biology and potential applications in biotechnology.

## Results

### Collagen glucosyltransferases form a dimer that binds UDP-glucose cooperatively

**Figure 1:**
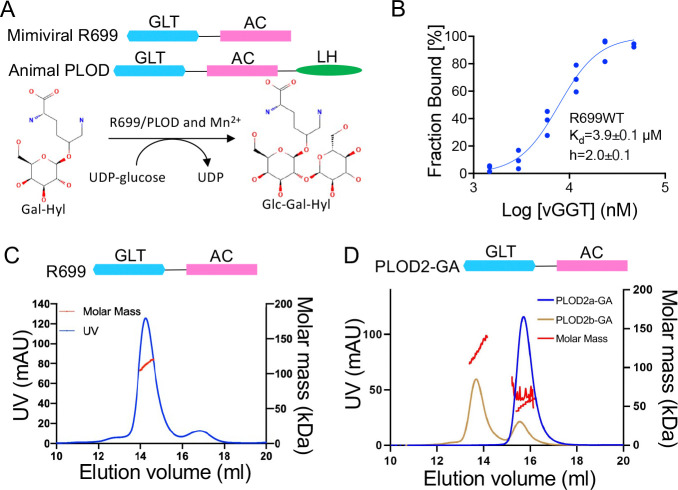
Mimiviral R699 binds UDP-glucose cooperatively and forms a dimer assembly similar to PLOD2b not PLOD2a. **A,** Schematic diagrams of mimiviral and human glucosyltransferase domain architecture (top) and glucosylation reaction (bottom). The glycosyltransferase (GLT), accessory (AC), and lysyl hydroxylase (LH) domains are highlighted. Both R699 and animal PLOD (procollagen-lysine 2-oxoglutarate 5-dioxygenase) catalyze the conversion of peptidyl galactosylhydroxylysine (Gal-Hyl) to glucosylgalactosylhydroxylysine (Glc-Gal-Hyl). **B,** R699’s UDP-glucose binding affinity and cooperativity were analyzed using microscale thermophoresis. The data represent the mean values obtained from triplicate measurements. **C and D,** Domain architecture and size exclusion chromatography with dynamic light scattering (SEC-DLS) of R699 (in C) and the human PLOD2 GLT-AC truncation (PLOD2-GA) of human PLOD2 isoforms (in D). On the basis of elution time (X-axis) and molar mass (Y-axis at the right), R699 (blue in C) and human PLOD2b truncation (PLOD2b-GA, light brown in D) form a dimer, whereas human PLOD2a truncation (PLOD2a-GA, blue in D) is monomeric.

We have recently identified a novel collagen galactosylhydroxylysyl glucosyltransferase or GGT activity in mimiviral R699 protein using enzymatic activity assays and mass spectrometric analyses (29). R699 contains two tandem Rossmann fold domains sharing moderate sequence similarity with human enzymes (Fig. 1A). Using microscale thermophoresis (MST), we determined that R699 binds UDP-glucose cooperatively (Fig. 1B, K_d_ = 3.9 ± 0.1 μM, Hill coefficient h = 2.0 ± 0.1). Competitive binding assays showed that unlabeled UDP-glucose competed with fluorescein-conjugated UDP-glucose for binding to R699, with an IC_50_ of 114 μM (Supplementary Fig. 1), suggesting that fluorescein is not involved in binding. The observed cooperativity and affinity of UDP-glucose binding recapitulate the findings on a recently identified collagen glucosyltransferase that is encoded by a collagen PLOD2 pre-mRNA alternatively spliced isoform b (PLOD2b) (9). Since a previous study indicated PLODs bind UDP-glucose at a 1:1 ratio, suggesting each collagen GGT has one UDP-glucose binding site (31), we thus hypothesized that collagen GGTs’ UDP-glucose binding cooperativity is due to enzyme oligomerization. We tested this hypothesis by analyzing the oligomeric states of R699 and PLOD2b using size exclusion chromatography and dynamic light scattering (SEC-DLS). Our results showed that both mimiviral R699 and human PLOD2b, but not PLOD2a, form dimers (Fig. 1C and D). These findings suggest that dimerization of collagen glucosyltransferases may underlie their cooperative binding of UDP-glucose.

### R699 forms an antiparallel dimer required for UDP-glucose binding cooperativity

**Figure 2:**
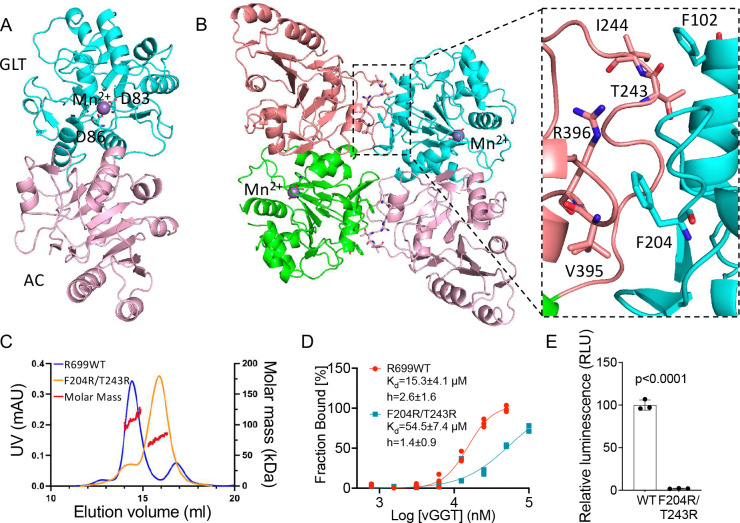
R699 dimerization is required for UDP binding affinity, cooperativity, and enzymatic activity. **A,** Ribbon diagram of the R699 bound to Mn^2+^ (royal purple). D83 and D86 coordinate the Mn^2+^ within the active site. **B,** Ribbon diagram of the R699 homodimer. The Mn^2+^ binding site is indicated, and the inset highlights key residues at the dimerization interface. **C,** SEC-DLS analysis of R699 wild type (R699WT in blue) and F204R/T243R mutant (orange). On the basis of elution time (X-axis) and molar mass (Y-axis at the right), R699WT forms a dimer, whereas the F204R/T243R mutant is monomeric. **D,** UDP-glucose binding affinity and cooperativity were measured using microscale thermophoresis for the samples described in C. The F204R/T243R mutation disrupts cooperative binding observed in the wild type. Data represent mean values from triplicate samples. **E,** Enzymatic activity assay of R699 using galactosylhydroxylysine as a substrate. The F204R/T243R mutant shows a complete loss of activity. Data represent mean values (± S.D.) from triplicate biological samples. Statistical significance was determined using a two-tailed Student’s t-test.

To elucidate the structural basis of collagen GGT dimerization, we solved the crystal structure of R699 in the presence of Mn^2+^. The structure was refined to a resolution of 1.80 Å, with diffraction data statistics and refinement details summarized in Supplementary Table 1. As anticipated, R699 consists of two tandem Rossmann fold domains arranged similarly to human GGT (Fig. 2A) (31). The overall structure, comprising the catalytic and adjacent accessory domains, shares moderate homology with human PLOD3 protein (PDB ID 6FXR), with a root-mean-square deviation (RMSD) of 3.1 Å. The catalytic domain of R699 shows much higher structural similarity to the human PLOD3 GGT catalytic domain (PDB ID 6FXR), with an RMSD of 1.5 Å (31), despite only 34% amino acid sequence identity. For comparison, a previously determined structure of the human PLOD3 GGT catalytic domain (PDB ID 6WFV) under different crystallization conditions aligns with the full-length PLOD3 protein structure (PDB ID 6FXR), with an RMSD of 0.9 Å. These findings establish R699 as a close structural homolog of human GGT.

We observed that two R699 molecules from an anti-parallel dimer in the asymmetric unit, occupying a surface area of ~1425 Å^2^ (Fig. 2B). This structural arrangement supports our hypothesis that UDP-glucose binding cooperativity arises from coordinated interactions between the two subunits within the dimer assembly. To investigate how the R699 dimer regulates UDP-glucose binding cooperativity, we generated a monomeric R699 mutant based on our structural insights and compared its UDP-glucose binding properties to the wild-type enzyme. Examination of the R699 dimer interface revealed key hydrophobic interactions stabilizing the dimer. These include contacts between F102 in one subunit and I244 and T243 in the opposite subunit (Fig. 2B inset). Additional hydrophobic interactions involve F204 in one subunit and V395 and R396 in the opposite subunit. Notably, these hydrophobic residues are not strictly conserved in PLOD2b (Supplementary Fig. 2), suggesting that the dimerization of PLOD2b is regulated differently. Consistent with the structural findings, SEC-DLS analyses showed that F204R/T243R double mutations caused R699 dimer loss (Fig. 2C). MST binding analyses and enzymatic activity assays further revealed that loss of dimerization resulted in a significant decrease in UDP-glucose binding affinity, abolished UDP-glucose binding cooperativity, and eliminated GGT enzymatic activity (Fig. 2D and E). To rule out the possibility that the F204R/T243R mutations caused protein misfolding, we performed circular dichroism spectroscopy, which showed no evidence of structural misfolding (Supplementary Fig. 3). These findings demonstrate that R699 dimerization is essential for UDP-glucose binding cooperativity and enzymatic activity.

### The structural basis of UDP-glucose binding cooperativity

**Figure 3:**
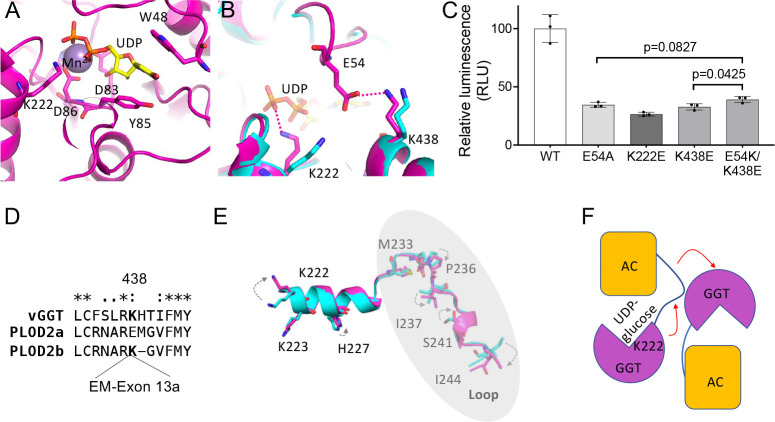
A two-state induced fit model of cooperative UDP-glucose binding. **A,** Ribbon diagram of the R699 active site bound to Mn2+ (royal purple) and UDP (yellow). The uridine ring of UDP is sandwiched between W48 and Y85. Additional key active site residues are labeled. **B.** Structural comparison of R699 with and without UDP bound, shown as cartoon diagrams. The UDP-bound structure is in purple, and the apo form is in cyan. Key active site residues undergoing conformational changes are highlighted, with UDP shown in yellow. **C.** Enzymatic activity assay of R699 using galactosylhydroxylysine as the substrate. Results are shown as mean values (± S.D.) from triplicate biological samples. *p* values were determined using two-tailed Student’s *t*-tests. Although *p* values between wild-type (WT) and mutant proteins are not displayed, they are <0.001. **D.** Sequence alignment of R699 with human PLOD2, highlighting K438 in R699 and its corresponding lysine residue in PLOD2 in bold. **E.** Ribbon diagram of the K222-containing helix and the dimer-stabilizing loop (highlighted within a gray oval) in R699. Structures with UDP bound (purple) and without UDP (cyan) are overlaid. Residues undergoing conformational changes are shown as sticks and labeled. **F.** Schematic model illustrating the proposed mechanism of positive cooperative interactions between the two active sites within the R699 dimer during UDP-glucose binding.

To investigate the basis of R699 UDP-glucose binding cooperativity, we co-crystallized R699 with UDP-glucose and Mn^2+^. The highest-resolution crystal diffracted to 1.75 Å, and the diffraction data were used to generate an electron density map via molecular replacement (Supplementary Table 2). The map allowed us to model UDP and Mn^2+^ into the structure, but the glucose moiety of UDP-glucose was not resolved (Fig. 3A). Comparative structural analyses of the UDP-bound and unbound forms revealed significant conformational changes in K222 upon UDP binding (Fig. 3B). In the unbound form, K222 points toward E54, positioning it within range for a potential salt bridge with E54. However, in this state, the electron density for E54 was absent, preventing precise modeling. Based on these findings, we speculate that, in the absence of UDP-glucose, E54 dynamically swings between K222 and K438, forming transient electrostatic interactions with these residues. In the UDP-bound form, K222 reorients to directly interact with the phosphate group of UDP-glucose in the R699 active site. Simultaneously, E54 forms a stable salt bridge with K438 in the accessory (AC) domain (Fig. 3B). These observations support an induced-fit model in which the “2K-1E triad” (K222, K438, and E54) undergoes conformational rearrangements upon UDP-glucose binding, optimizing the active site for catalysis. To probe the functional roles of this triad, we generated a targeted mutation E54A to eliminate both salt bridge options as well as K222E or K438E to disrupt individual salt bridges. Enzymatic activity assays revealed that all mutations severely impaired R699’s activity (Fig. 3C). Interestingly, the E54K/K438E double mutant, designed to restore a salt bridge between residues 54 and 438, showed slightly higher enzymatic activity than the single E54A or K438E mutants. These findings suggest that the E54-K438 salt bridge facilitates UDP binding, consistent with our structural data. To exclude the possibility that these mutations caused protein misfolding, we analyzed the mutants using circular dichroism spectrometry. The results showed no significant differences between the mutants and the wild-type protein (Supplementary Fig. 4), confirming that the loss of activity was not due to misfolding. These findings provide mechanistic insights into the role of the 2K-1E triad in R699’s UDP-glucose binding and enzymatic function.

Interestingly, sequence analysis showed that K438 is present exclusively in PLOD2b but not in PLOD2a protein (Fig. 3D). K222 is strictly conserved in PLOD2b protein, while E54 may be substituted by a similar acidic residue, aspartate (Supplementary Fig. 2). These observations suggest that the 2K-1E triad is conserved in PLOD2b protein and may contribute to its unique UDP-glucose binding cooperativity. Structural analysis revealed that conformational changes in the active site upon UDP-glucose binding are accompanied by conformational changes in a loop connecting the GGT and AC domains. The residue in the loop that undergoes a major conformational change is I244 (Fig. 3E), which plays a key role in stabilizing the R699 dimer (Fig. 2B inset). Since the loop resides between the two opposite active domains, it may play a role in transmitting UDP-glucose binding signals between the two domains, thereby promoting positive cooperativity (Fig. 3F). These findings support a two-state induced-fit model for UDP-glucose binding cooperativity.

### The mechanism of collagen binding

**Figure 4:**
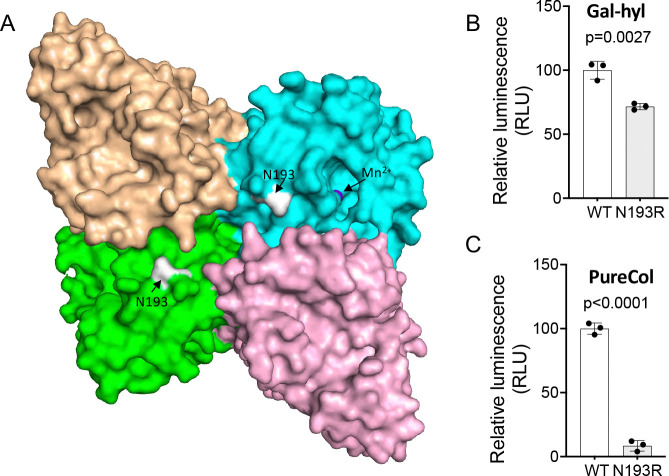
R699 dimer creates a continuous cleft that may be involved in collagen binding. **A.** Surface representation of the R699 homodimer. The dimerization interface creates a continuous cleft flanked by the active sites. One of 2 Mn^2+^ atoms (slate) is shown within an active domain (cyan), and both N193 (white) residues are highlighted within the cleft. **B.** Enzymatic activity assay of R699 using galactosylhydroxylysine (Gal-Hyl) as a substrate. Results are expressed as mean values (± S.D.) from triplicate biological samples. **C.** Enzymatic activity assay of R699 using denatured bovine type I collagen (PureCol) as a substrate. Results are also expressed as mean values (± S.D.) from triplicate biological samples. For both panels, *p* values were determined using two-tailed Student’s *t*-tests.

Our structural data indicate that the R699 dimer assembly creates a continuous U-shaped surface cleft flanked by two active sites (Fig. 4A), suggesting that this cleft may function as a collagen-binding site. To investigate this possibility, we chose residue N193 located at the center of the cleft (Fig. 4A) and introduced an N193R mutation. The N193R mutant and wild-type proteins were expressed, purified, and subjected to enzymatic activity assays. The N193R mutation had minimal impact on R699’s activity toward galactosylhydroxylysine (Fig. 4B) but significantly reduced its activity toward collagen (Fig. 4C). Further analyses using circular dichroism suggested that N193R was not deleterious to protein folding (Supplementary Fig. 5). These results support a role of the surface cleft in binding large peptidyl substrates.

### The basis of disaccharide binding

**Figure 5:**
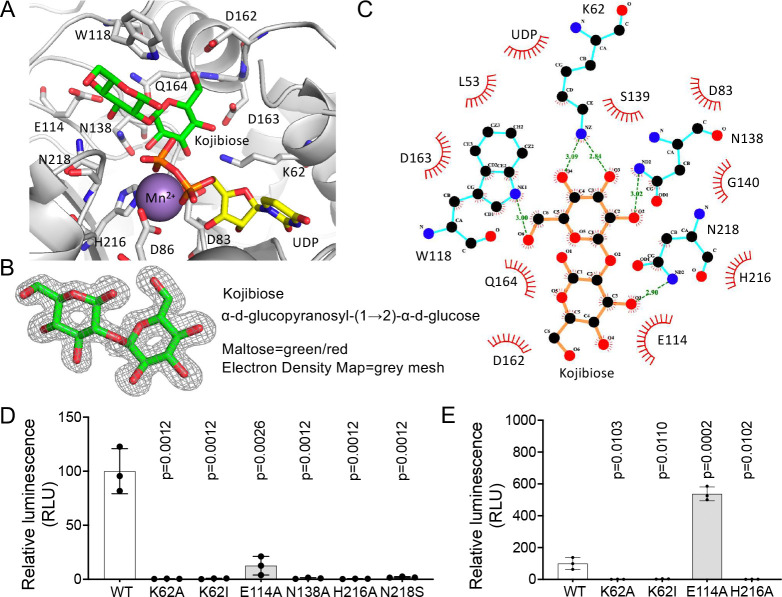
kojibiose engages a network of interactions in the R699 active site. **A, A.** Ribbon diagram of the R699 active site highlighting key components, including Mn^2+^ (royal purple), UDP (yellow), and kojibiose (green). Critical residues involved in substrate interaction are labeled. **B.** Kojibiose (green/red) was modeled on the basis of the electron density map (gray mesh, contour level σ = 1), demonstrating its fit. **C.** LIGPLOT+ diagram illustrating the interaction network of kojibiose within the R699 active site. Key hydrogen bonds and hydrophobic interactions are highlighted, demonstrating the structural basis for substrate binding. **D.** Enzymatic activity assay of R699 using galactosylhydroxylysine as the substrate. Data represent mean values (± S.D.) from triplicate biological samples. **E.** Measurement of UDP-glucose hydrolysis by R699 using a luciferase-based assay. Data are presented as mean values (± S.D.) from triplicate biological samples. For panels D and E, *p* values were determined using two-tailed Student’s *t*-tests.

To elucidate the structural basis of collagen glucosyltransferase substrate binding, we co-crystallized R699 with UDP-glucose and galactosyl Hyl. Despite screening numerous crystals, we were unable to obtain diffraction data with sufficient galactosyl Hyl electron density. Consequently, we explored R699 co-crystallization with monosaccharides like glucose and galactose as sugar acceptor mimics. Surprisingly, although glucose and galactose were added in equal amounts for crystallization, electron density at the active site revealed bound UDP and kojibiose (α-d-glucopyranosyl-(1→2)-α-d-glucose) at 1.50 Å (Fig. 5A and B, Supplementary Table 3). These findings indicate that R699 transfers the glucose moiety from UDP-glucose to a glucose acceptor, generating kojibiose-a product distinct from the 4’-epi-kojibiose found on animal collagens.

Our structural analysis showed that the first glucose of kojibiose occupies a pocket between Mn^2+^ and D163/Q164 (Fig. 5A). O2 of this glucose interacts with the ND2 of N138 and NE2 of H216 (Fig. 5C), while O3 and O6 form hydrogen bonds with K62 and W118/D162, respectively (Fig. 5C). Sequence alignment revealed that these residues are highly conserved, underscoring their importance in substrate recognition (Supplementary Fig. 2). Mutagenesis studies further confirmed their roles, as substitutions of these residues resulted in significant catalytic activity loss (Fig. 5D). The catalytic roles of W118 and the polyacidic residues (D162 and D163) have been investigated in previous studies (29,31,32), providing additional support for their essential contributions to enzymatic function. These findings provide novel insights into R699’s substrate binding and catalytic activity.

The second glucose is positioned between W118 and a loop, forming extensive interactions with UDP and E114 of R699 (Fig. 5A). The O3’ hydroxyl group of the second glucose interacts with the ND2 atom of N218 (Fig. 5C). Both E114 and N218 are critical for R699 catalysis, as demonstrated by site-directed mutagenesis and enzymatic activity assays (Fig. 5D). While both residues are highly conserved across species (Supplementary Fig. 2), E114 is uniquely essential for collagen glucosylation by LH3, whereas N218 is not strictly required. Interestingly, N218 is the closest residue to the O1 hydroxyl group of the first glucose, located approximately 5 Å away, and 6.5 Å from the diphosphate moiety (Supplementary Fig. 6). These findings suggest that no general base is positioned close enough to directly participate in catalysis, consistent with previous reports that collagen GGTs are retaining-type glycosyltransferases (33). Additionally, R699 exhibits moderate uncoupling of UDP-glucose hydrolysis in the absence of an acceptor substrate, a property similar to human LH3. Our results suggest that E114 plays a specific role in acceptor glucosylation rather than uncoupling UDP-glucose hydrolysis, consistent with its proposed function in binding the acceptor substrate (Fig. 5E). In contrast, residues essential for interactions with the first sugar moiety, such as K62 and H216, are critical for both coupling and uncoupling UDP-glucose hydrolysis (Fig. 5E). Circular dichroism spectrometry confirmed that none of the mutants exhibited signs of misfolding, ensuring that the observed effects are not attributable to structural instability (Supplementary Fig. 7).

### R699 has kojibiose synthase activity

**Figure 6:**
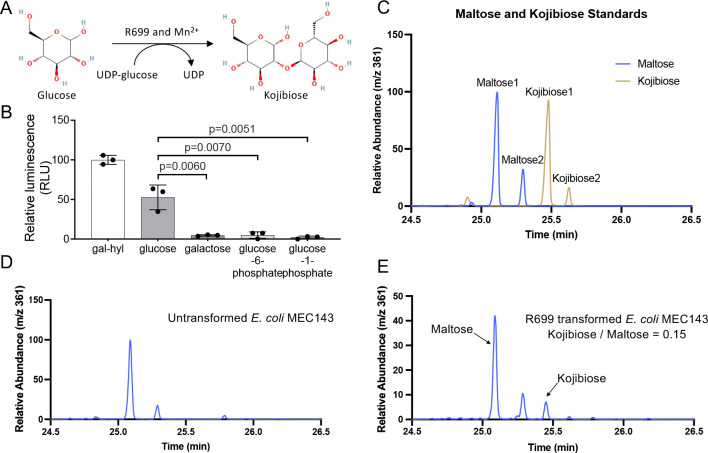
R699 has kojibiose synthase activity. **A.** Schematic representation of the proposed kojibiose synthesis reaction catalyzed by R699. **B.** Enzymatic activity assay of R699 using various substrates, including galactosylhydroxylysine (Gal-Hyl), glucose, galactose, glucose-6-phosphate, and glucose-1-phosphate. Data are presented as mean values (± S.D.) from triplicate biological samples. *p* values were calculated using two-tailed Student’s *t*-tests. **C, D, and E.** Gas chromatograms of standards (in C) and cell lysates from untransformed E. Coli strain MEC143 (in D) versus MEC143 transformed with R699 (in E). Chromatograms were generated by extracting m/z 361, a characteristic ion of methyloximated, 8-trimethylsilyl derivatized disaccharides. The positions of maltose and kojibiose major peaks are marked in E. Quantitative analysis using the major peaks indicates that kojibiose yield is approximately 15% of maltose, the most abundant disaccharide in *E. coli*. Note there are two isomers of derivatized disaccharides.

To confirm R699’s substrate specificity, we analyzed its activity using UDP-glucose as the sugar donor and either glucose or galactose as the sugar acceptor, with galactosyl Hyl serving as a positive control. The results showed that R699 exhibited robust activity toward glucose but no detectable activity with galactose (Fig. 6A and B), supporting R699’s kojibiose synthase activity. To evaluate R699’s potential kojibiose synthase activity *in vivo*, we transformed and overexpressed R699 in the *E. coli* BL21 strain and analyzed the products using gas chromatography-mass spectrometry (GC-MS). However, kojibiose was undetectable. We reasoned that the absence of kojibiose production might be due to low intracellular glucose levels, as glucose is rapidly phosphorylated by hexokinases upon entering *E. coli* cells. Based on these findings, we hypothesized that R699 is unable to modify phosphorylated glucose substrates, such as glucose-6-phosphate or glucose-1-phosphate. To test this hypothesis, we performed enzymatic activity assays using phosphorylated glucose as potential sugar acceptors. The results confirmed that R699 could not glycosylate glucose-6-phosphate or glucose-1-phosphate, supporting the hypothesis that R699 requires unphosphorylated glucose as its substrate. To address the issue of low intracellular glucose levels, we expressed R699 in the *E. coli* MEC143 strain (Supplementary Fig. 8), which is deficient in hexokinase activity and thus accumulates higher levels of unphosphorylated glucose. GC-MS analysis identified peaks unique to kojibiose exclusively in MEC143 cells expressing R699 (Fig. 6C–E, and Supplementary Fig. 9). The identity of the major kojibiose peak was further confirmed by GC-MS analysis of chemical standards (Supplementary Fig. 10). Quantitative analysis indicated that kojibiose yield was approximately 15% relative to maltose (Fig. 6E and Supplementary Fig. 10 and 11), the most abundant disaccharide in E. coli. These findings demonstrate that kojibiose production occurred exclusively in MEC143 cells expressing R699. These findings also demonstrate that R699 functions as a kojibiose synthase, specifically utilizing unphosphorylated glucose as its substrate.

## Discussion

We found that both mimiviral and human collagen glucosyltransferases bind UDP-glucose cooperatively. This cooperative binding suggests that these enzymes can sharply adjust their collagen glucosylation activity in response to changes in UDP-glucose levels. Such regulation may be critical during mimivirus-host interactions, where fluctuations in UDP-glucose levels occur, and during fibroblast differentiation (21), a process requiring precise collagen modifications. These findings highlight a potential conserved mechanism by which collagen glucosyltransferases respond dynamically to variations in their substrate availability.

The primary hosts of mimiviruses are amoebae and other protists (34). The amoebae life cycle consists of two main stages: an active trophozoite stage and a dormant cyst stage. The latter enables amoebae to survive for days to weeks in external environments (35). During the transition from trophozoite to cyst, UDP-glucose levels in amoebae increase fivefold (30), potentially influencing the activity of UDP-glucose-dependent enzymes. Our findings demonstrated that mimiviral collagen glucosyltransferase binds UDP-glucose cooperatively, suggesting that mimiviral glucosyltransferase activity and collagen glucosylation are highly sensitive to changes in UDP-glucose levels. This implies that amoebae encystment may sharply upregulate mimiviral collagen glucosylation by elevating UDP-glucose concentrations. However, previous studies indicate that mimivirus infects amoebae during the trophozoite stage, not during the cyst stage (36,37). Furthermore, mimiviral infection has been shown to prevent amoebae encystment (37), leaving the virus within trophozoites where intracellular UDP-glucose levels are relatively low. These observations may explain why determining mimiviral collagen glycosylation has been challenging. These findings also raise intriguing questions about the specific roles of mimiviral collagen glycosylation in amoebae encystment and mimivirus-host interactions, warranting further investigation into this complex relationship.

In animal cells, intracellular UDP-glucose levels are tightly regulated. It has been reported that downregulation of UDP-glucose promotes mesenchymal differentiation, as UDP-glucose facilitates SNAIL1 mRNA stabilization by binding to Hu antigen R (38). As a result, UDP-glucose levels are low in mesenchymal cells, such as fibroblasts. Paradoxically, fibroblasts are the major collagen producers and require UDP-glucose for collagen glucosylation. These findings raise the question of how collagen glucosylation occurs in the presence of low UDP-glucose levels. Recent research, including our own, has shown that mesenchymal differentiation upregulates PLOD2b (21), which binds UDP-glucose tightly and cooperatively (9). These findings suggest that the cooperative binding of UDP-glucose enables the tight regulation of collagen glucosyltransferase activity and collagen glucosylation during mesenchymal differentiation. Alternatively, high PLOD2b levels could deplete UDP-glucose during collagen production in fibroblasts, as collagen biosynthesis-being the most abundant protein in mammals-requires significant cellular resources. If this hypothesis is correct, it implies that collagen production may reduce UDP-glucose levels to stabilize SNAIL1 mRNA and reinforce the mesenchymal cell fate.

We present a comprehensive set of collagen glucosyltransferase crystal structures: 1) with Mn^2+^, 2) with Mn^2+^ and UDP, and 3) with Mn^2+^, UDP, and disaccharide product. These structures reveal key features and conformational changes involved in substrate binding and cooperativity. Our study provides critical insights into collagen glucosyltransferase function, which could inform the development of antagonists for collagen glucosylation-associated diseases, such as cancer and fibrosis. Additionally, these insights may aid in engineering collagen glucosyltransferases to produce recombinant collagen and collagen peptides for biomedical research and applications.

The structures we presented here identify a continuous long cleft with two flanking active sites, suggesting this cleft serves as a collagen-binding site. Based on the size of the cleft, we reasoned that R699 recognizes a single collagen alpha chain, not a triple helix, which is consistent with its enzymatic activity toward denatured collagen. Interestingly, collagen lysyl hydroxylases form a similar collagen-binding cleft at the dimer interface (28), indicating that collagen-modifying enzymes recognize their substrate in a similar manner. The two active sites are located in an anti-parallel orientation, which could allow them to bind independently to two unzipped collagen chains or a single collagen chain via looping during collagen biosynthesis.

We crystallized R699 with an equal molar ratio of glucose and galactose as sugar acceptor. The structures presented here suggests that R699 prefers to glycosylate glucose over galactose, forming kojibiose. Kojibiose was initially isolated from Koji extract and was also found in low levels in honey (39,40). It has been shown that kojibiose is resistant to be fermented in the mouth or absorbed in small intestine but supports the growth of healthy gut microbiome, thus, it is beneficial to manage dental caries and blood sugar surges as well as promote gut health (41,42). However, industrial production of kojibiose remains challenging due to the lack of low-cost and efficient production methods (43). Our serendipitous results suggest that R699 is a potential kojibiose synthase, capable of producing kojibiose in a manner similar to sucrose production in plants. These findings open the possibility of engineering R699 and integrating this simple kojibiose synthesis step into sugar-producing microorganisms, such as the baker’s yeast, for industrial production of kojibiose.

Additionally, our findings suggest that R699 may prefer glucosylhydroxylysine over galactosylhydroxylysine as a sugar acceptor. Consistent with these results, a previous report indicated that mimiviral L230 glycosylates hydroxylysine to glucosylhydroxylysine or glucosyl Hyl (27). Together, these findings propose a testable model in which certain Lys residues in mimiviral collagen are hydroxylated and glucosylated one or two times to form glucosyl Hyl and glucosylglucosyl Hyl. This is distinct from human collagen, which contains galactosyl Hyl and glucosylgalactosyl Hyl. Furthermore, since we also found that R699 can modify galactosyl Hyl-containing collagen peptides (29), our results suggest that R699 is a multifunctional enzyme.

## Methods

### Cloning, Protein Expression and Purification

The R699 gene was synthesized by Genscript and cloned into a modified version of the pET28 vector using BamH1 and EcoR1 sites for enzymatic activity assays. This modified vector replaced the thrombin recognition site with PreScission and BamH1 recognition sites, with the endogenous BamH1 site being eliminated. Mutant constructs were generated using Agilent’s QuickChange Lightning Site-Directed Mutagenesis Kit. R699 was cloned into a version of the pET28-mCherry vector for crystallization purposes, employing BamH1 and EcoR1 sites. This vector contains the mCherry gene sequence and a PreScission recognition site inserted between the Nhe1 and BamH1 sites. To express R699 in an *E. coli* strain that lacks T7 RNA polymerase, His6-R699 fusion was synthesized by Gene Universal and cloned into pMAL-C4X vector using NdeI and SalI sites. PLOD2-GA truncations were optimized with the PROSS server to improve their expression in *E. coli* (44)*.* The sequences of PLOD2-GA truncations are shown in Supplementary Fig. 12.

All plasmids were verified through Sanger sequencing and then transformed into *E. coli* strain BL21 DE3 (NEB) for protein expression. A small-scale BL21 overnight culture with 50 mg per liter of kanamycin (GoldBio) was prepared, and 10 ml of this culture was used to inoculate an 800 ml large-scale culture using Terrific Broth Medium (Alpha Biosciences), with the same amount of kanamycin. The culture was grown at 37°C until reaching an OD600 of 1.5, then induced with 1 mM isopropyl β-D-1-thiogalactopyranoside (IPTG, GoldBio), and finally grown at 16°C for 18 hours. After growth, cells were collected, pelleted, and resuspended in binding buffer (20 mM Tris, pH 8.0, 200 mM NaCl, and 15 mM imidazole). Following cell lysis by sonication, the lysate was centrifuged at 23,000g for 15 minutes. The recombinant R699 proteins (wild type or mutants) were purified using immobilized metal affinity chromatography and eluted with elution buffer (200 mM NaCl and 300 mM imidazole, pH 8.0). R699 protein was dialyzed at 16°C for 18 hours in 20 mM HEPES, pH 7.4, and 150 mM NaCl for enzymatic activity assays.

To test kojibiose synthesis *in vivo*, pMAL-C4X-His6-R699 was expressed in hexokinase-deficient *E. coli* strain MEC143, a generous gift from Dr. Mark A. Eiteman from the University of Georgia (45). Ten ml of R699 transformed MEC143 overnight culture was used to inoculate an 800 ml large-scale culture of Miller LB Broth (Midland Scientific) supplemented with 100 mM glucose (MilliporeSigma) and 90 μM MnCl_2_ (MilliporeSigma). The culture was grown at 37°C until reaching an OD600 of 1.5, then induced with 1 mM IPTG at 37°C for 18 hours. R699 was purified using immobilized metal affinity chromatography, eluted with elution buffer, concentrated to 0.5 mg ml^−1^, separated and visualized using SDS-PAGE and Bio-Safe^™^ Coomassie Stain (Bio-Rad). MEC cell lysates were also analyzed using GC-MS to detect Kojibiose.

### GC-MS Analysis

Bacterial pellets were lysed by brief sonication in −20°C 80% methanol followed by incubation at −20°C for 1 hour to precipitate proteins. Lysates were centrifuged at 17,000 *g* for 10 minutes at 4°C and supernatants were transferred to 1.5 ml polypropylene tubes. For GC-MS derivatization, lysates were dried on a Centrivap (Labconco) followed by the addition of 50 μl 20 mg ml^−1^ methoxyamine (Sigma) in pyridine (Thermo) and incubated at 30°C for 90 minutes. Samples were centrifuged at 17,000 *g* for 10 minutes at room temperature and 40 μl was transferred to a glass GCMS vial where 80 μl of N-Methyl-N-(trimethylsilyl)trifluoroacetamide (MSTFA) + 1% Chlorotrimethylsilane (TCMS) (Thermo) was added to each sample followed by incubation at 37°C for 30 minutes. For maltose and kojibiose standards, 20 nmol of each standard was dried and derivatized identically to cell extracts.

For GC-MS analysis, 1 μl of each sample or standard was injected into an Agilent 5977b GC-MS containing an Agilent HP-5MS column. The inlet was set at 250°C with a pressure of 4.9 psi and septum purge flow of 3 ml min^−1^. Splitless inlet mode was set to 10.5 ml min^−1^ at 1 minute. Initial oven temperature was set to 60°C for 1 minute, followed by a linear ramp to 325°C at a rate of 10°C min^−1^. Full-scan MS detection range was set to 50–550 m/z with a source and quad temperature of 230°C and 150°C, respectively. Data files were imported into MassHunter Qualitative Analysis (Agilent) for peak and mass spectrum extraction. MassHunter chromatograms were exported as text files and imported into GraphPad Prism to generate figures.

### Crystallization, Structure Determination, and Refinement

mCherry-R699 was first purified using immobilized metal affinity chromatography, as described above. The eluted recombinant protein was cleaved with PreScission protease at 4°C for 18 hours while dialyzing in gel filtration buffer (20 mM Tris, pH 8.0, 200 mM NaCl). After PreScission protease cleavage, R699 was purified again using reverse immobilized metal affinity chromatography to remove mCherry protein and other contaminants that bind to nickel resin. The eluted protein was further purified by gel filtration using a Hiload 16/60 Superdex 200 PG column at a flow rate of 1 ml per minute. Peak fractions were combined and concentrated for crystal trials. High-quality crystals were obtained via hanging drop vapor diffusion using a Mosquito liquid handling robot (TTP Labtech) with a 200-nL drop. For R699 with Mn^2+^ structure, R699 (30 mg ml^−1^), supplemented with 2 mM manganese(II) chloride, was mixed with 200 mM ammonium formate, 20% (w v^−1^) PEG 3350 at a 1:1 ratio and incubated at 18°C. For R699 with Mn^2+^ and UDP structure, R699 (12 mg ml^−1^), supplemented with 10 mM manganese(II) chloride, 10 mM UDP-glucose, and 50% glycerol, was mixed with 180 mM ammonium chloride, 18% (w v^−1^) PEG 3350, and 4% (v v^−1^) 1-Propanol at a 1:1 ratio and incubated at 18°C. For R699 with Mn^2+^, UDP, and kojibiose structure, R699 (28 mg ml^−1^), supplemented with 10 mM manganese(II) chloride, 10 mM UDP-glucose, 0.15 g ml^−1^ glucose, and 0.15 g ml^−1^ galactose, was mixed with 100 mM HEPES pH 7.0, 10% (w v^−1^) PEG 6000 at a 1:1 ratio and incubated at 18°C. Diffraction data were collected on the 22-ID beamline of SERCAT at the Advanced Photon Source, Argonne National Laboratory (Supplementary Table. 1–3) at 110K with a wavelength of 1.0 Å. Data were processed using XDSgui and DIALS included in CCP4 (46–49). An initial R699 crystal structure was solved by molecular replacement with Phenix using RoseTTAFold and AlphaFold models as search templates (50,51). The refined individual domains were then used as search models for solving the other R699 crystal structures. The structures were then fully built and refined iteratively using Coot and Phenix (52–54), respectively. Protein structure similarity was compared using the Dali server (55,56), while structure interface analysis was performed using the protein interfaces, surfaces, and assemblies’ service PISA at the European Bioinformatics Institute (57). The initial cartoons of disaccharides in the R699 active site were constructed using Ligplot+ (58). Molecular graphics were prepared using Pymol.

### SEC-DLS Analysis

The molar mass of R699 and human PLOD2 proteins were analyzed by size exclusion chromatography and dynamic light scattering analyses (SEC-DLS). Superdex 200 (10/300) was equilibrated with SEC-DLS buffer (20 mM Tris, pH 8, 200 mM NaCl) at 4°C overnight using Akta purifier (GE Healthcare) and connected downstream to dynamic light scattering detector (miniDAWN TREOS) and refractive index (RI) (Optilab T-rEX) instruments (Wyatt Technology). Proteins (3 mg ml^−1^, 150 μl) were injected into the Superdex 200 column with a flow rate of 0.5 ml min^−1^ and the DLS data were analyzed by ASTRA 7.1 program. The results were from a single biological sample. Each protein was analyzed once.

### GGT Enzymatic Activity Assay

GGT activity was measured using a method previously described (9). The assay was conducted in reaction buffer (100 mM HEPES buffer pH 8.0, 150 mM NaCl) at 37°C for 1 hour with 1 μM R699 enzyme, 100 μM MnCl_2_, 200 μM UDP-glucose (MilliporeSigma), 1 mM dithiothreitol, and 1.75 mM galactosyl hydroxylysine (Gal-Hyl, MedChemExpress) or 2 μM PureCol^®^ (Advanced BioMatrix). PureCol^®^ was denatured at 95°C for 5 minutes and chilled on ice immediately before use. For UDP-glucose hydrolysis assay, no sugar acceptor was added. Following the manufacturer’s instructions, GGT activity was measured by detecting UDP production with an ATP–based luciferase assay (UDP-Glo^™^ Glycosyltransferase Assay, Promega). Experiments were performed in triplicate from distinct samples, and an unpaired Student’s t-test was used to compare the enzymatic activity of different samples.

### Microscale Thermophoresis

To conduct microscale thermophoresis, glucose-UDP-(PEG)6-fluorescein conjugate (10 μl at 50 nM) was mixed with an equal volume of serially diluted unlabeled R699 protein in 20 mM Tris, pH 8.0, 200 mM NaCl, 5 mM Mn^2+^, and 0.05% Tween-20. After incubation at 25°C for 15 minutes, the samples were loaded into silica capillaries (Nanotemper Technologies). For the competition assay, fixed concentrations of glucose-UDP-(PEG)6-fluorescein conjugate (50 nM) and R699 (20 μM) were titrated with different concentrations of unlabeled UDP-glucose. For Fig 1B, glucose-UDP-(PEG)6-fluorescein conjugate was purchased from MilliporeSigma (Cat # SMB00284) and data collection was performed at 20°C using Dianthus NT23.Pico (Nanotemper Technologies). For Fig. 2D and Supplementary Fig. 1, glucose-UDP-(PEG)6-fluorescein conjugate was purchased from AAT Bioquest (Cat # 11706) and data collection was performed at 20°C using Monolith NT.115 (Nanotemper Technologies). Curves were analyzed using Prism 10 to fit K_d_ according to the law of mass action and to determine IC_50_. The experiment was repeated in triplicates unless stated otherwise, and the results represent the mean values from repeated biological samples.

## Supplementary Material

Supplement 1

## Data Availability

Crystal structures have been deposited in the Worldwide Protein Data Bank under RCSB accession ID numbers 9DYT, 9DZS, and 9E92.
