# Supplementary material for: Structural basis of collagen glucosyltransferase function and its serendipitous role in kojibiose synthesis
Source: Res Sq. 2025 Jan 29:rs.3.rs-5850681. Preprint. [Version 1] doi: 10.21203/rs.3.rs-5850681/v1 (PMC11838735; doi:10.21203/rs.3.rs-5850681/v1)
Supplement: Supplement 1 [file NIHPPrs5850681v1-supplement-1.pdf]

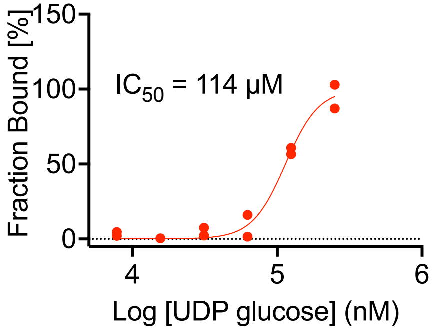

Figure 1s: UDP-glucose competitive binding assay determined by microscale thermophoresis. Fixed concentrations of fluorescein-conjugated UDP-glucose (50 nM) and R699 (20 μM) were titrated with different concentrations of unlabeled UDP-glucose to generate the curve. The curve was used to calculate the IC_{50}. Results are mean values from duplicated samples (n=2).

```
Mimi-R699       ----------------------------MEQSNNDDNLLVLGIGISVHKTDGVLRFEKY 31
hPLOD3          MTSSGPGPRFLLLLPLLLPPAASASDRPRGRDPVNPEKLLVI--TVATAETEGYLRFLRS 58
hPLOD1          -----MRPLLL---LALLGWLLL---AEAKGDAKPEDNLLVL--TVATKETEGFRRFKRS 47
hPLOD2b         MGGCTVKPQLLLLALVLHPWNPCLGADSEKPSSIPTDKLLVI--TVATKESDGFHRFMQS 58
                               .    ::***:   ::. :::*  ** :

Mimi-R699       CQAHNLQYMIVGEGKKWNGGNLESEAGGGQKINELLIALESIKD--NKLIVVCDTYDLIP 89
hPLOD3          AEFFNYTVRTLGLGEEWRGGDVARTVGGGQKVRWLKKEMEKYADREDMIIMFVDSYDVIL 118
hPLOD1          AQFFNYKIQALGLGEDWNVEKGT-SAGGGQKVRLLKKALEKHADKEDLVILFTDSYDVLF 106
hPLOD2b         AKYFNYTVKVLGQGEEWRGGDGINSIGGGQKVRLMKEVMEHYADQDDLVVMFTECFDVIF 118
                .: .*     :* *:.*.  .     *****:. :   :*   *  : :::. : :*::

Mimi-R699       LSGPEEILRKYRFLTPDNKVVFSSELYCWPDASLVERYPKVDTKYKYLNSGAFMGYRDDI 149
hPLOD3          AGSPTELLKKFV--QSGSRLLFSAESFCWPEWGLAEQYPEVGTGKRFLNSGGFIGFATTI 176
hPLOD1          ASGPRELLKKFR--QSRSQVVFSAEELIYPDRRLETKYPVVSDGKRFLGSGGFIGYAPNL 164
hPLOD2b         AGGPEEVLKKFQ--KANHKVVFAADGILWPDKRLADKYPVVHIGKRYLNSGGFIGYAPYV 176
                 ..* *:*:*:       :::*:::   :*:  *  :** *    ::*.**.*:*:   :

Mimi-R699       YEMIKN-GVKDRDDDQLFFSIKFIETD-----KIVLDYKCELFQAMYRCNSDLVVH---- 199
hPLOD3          HQIVRQWKYKDDDDDQLFYTRLYLDPGLREKLSLNLDHKSRIFQNLSGALDEVVLKFDRN 236
hPLOD1          SKLVAEWEGQDSDSDQLFYTKIFLDPEKREQINITLDHRCRIFQNLDGALDEVVLKFEMG 224
hPLOD2b         NRIVQQWNLQDNDDDQLFYTKVYIDPLKREAINITLDHKCKIFQTLNGAVDEVVLKFENG 236
                 .:: :   :* *.****::  :::       .: **::..:** :  . .::*::

Mimi-R699       KNRIFNGYTNSYPVFAHGNGPAKKLLNHMEGYFMTEPIDGSSN--------TINTFKLDN 251
hPLOD3          RVRIRNVAYDTLPIVVHGNGPTKLQLNYLGNYVPNGWTPEGGCGFCNQDRRTLPGG--QP 294
hPLOD1          HVRARNLAYDTLPVLIHGNGPTKLQLNYLGNYIPRFWTFETGCTVCDEGLRSLKGIGDEA 284
hPLOD2b         KARAKNTFYETLPVAINGNGPTKILLNYFGNYVPNSWTQDNGCTLCEFDTVDLSAV--DV 294
                : *  *   :: *:  :****:*  **:: .*.        .          :     :

Mimi-R699       EPKVFFALYVDSNDLSALKQFLGKVASIQYGNKVIYLYDRSDNEQNRKLIQISYPNYHT- 310
hPLOD3          PPRVFLAVFVEQPT-PFLPRFLQRLLLLDYPPDRVTLFLHNNEVFHEPHIADSWPQLQDH 353
hPLOD1          LPTVLVGVFIEQPT-PFVSLFFQRLLRLHYPQKHMRLFIHNHEQHHKAQVEEFLAQHGSE 343
hPLOD2b         HPNVSIGVFIEQPT-PFLPRFLDILLTLDYPKEALKLFIHNKEVYHEKDIKVFFDKAKHE 353
                 * * ..::::.     :  *:  :  :.*  . : *: :..:  :.  :     :

Mimi-R699       ---------------GVTKYVFDDFK--KSDAQFYFLLEQNCIITKKDILHELIMQVKDN 353
hPLOD3          FSAVKLVGPEEALSPGEARDMAMDLCRQDPECEFYFSLDADAVLTNLQTLRIL---IEEN 410
hPLOD1          YQSVKLVGPEVRMANADARNMGADLCRQDRSCTYYFSVDADVALTEPNSLRLL---IQQN 400
hPLOD2b         IKTIKIVGPEENLSQAEARNMGMDFCRQDEKCDYYFSVDADVVLTNPRTLKIL---IEQN 410
                               . :: :  *:   . .. :** :: :  :*:   *: *   :::*

Mimi-R699       HRVISPMIGYEQNSTRTNFWGDI-EDGYYKRSENYLDLAKHKVRGLWNVPYVYGVILMHE 412
hPLOD3          RKVIAPMLSR-HGKLWSNFWGALSPDEYYARSEDYVELVQRKRVGVWNVPYISQAYVIRG 469
hPLOD1          KNVIAPLMTR-HGRLWSNFWGALSADGYYARSEDYVDIVQGRRVGVWNVPYISNIYLIKG 459
hPLOD2b         RKIIAPLVTR-HGKLWSNFWGALSPDGYYARSEDYVDIVQGNRVGVWNVPYMANVYLIKG 469
                :.:*:*::   :.   :**** :  * ** ***:*:::.: .  *:*****:    :::

Mimi-R699       SVVRN----WDLSMVKYNDKDMDLCFSLRK--------------------HTIFMYMIN 447
hPLOD3          DTLRMELPQRDVFSGSDTDPDMAFCKSFRD--------------------KGIFLHLSN 508
hPLOD1          SALRGELQSSDLFHHSKLDPDMAFCANIRQ--------------------QDVFMFLTN 498
hPLOD2b         KTLRSEMNERNYFVRDKLDPDMALCRNAREMTLQREKDSPTPETFQMLSPPKGVFMYISN 529
                ..:*      :    .  * ** :* . *.                     : :*:.: *

Mimi-R699       NNNYGYMV---------------------------------------------------- 455
hPLOD3          QHEFGRLLATSRYDTEHLHPDLWQIFDNPVDWKEQYIHENYSRALEGEGIVEQPCPDVYW 568
hPLOD1          RHTLGHLLSLDSYRTTHLHNDLWEVFSNPEDWKEKYIHQNYTKALAG-KLVETPCPDVYW 557
hPLOD2b         RHEFGRLLSTANYNTSHYNNDLWQIFENPVDWKEKYINRDYSKIFTE-NIVEQPCPDVFW 588
                .:  * ::
```

Figure 2s: Sequence alignment of mimiviral R699 and human collagen glucosyltransferases. Dimer interface residues highlighted in blue, 2K-1E triad in green, and sugar-binding residues in yellow.

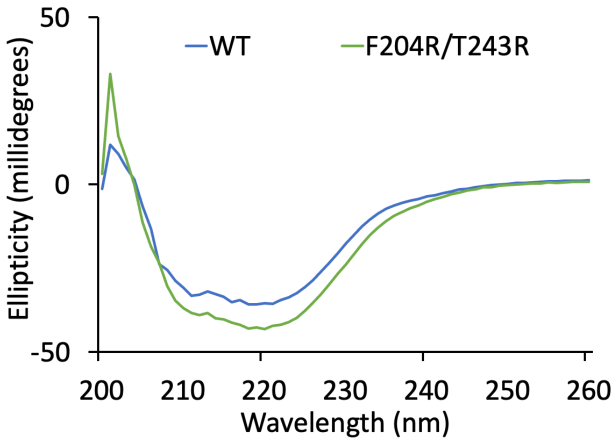

Figure 3s: Circular dichroism spectra of R699 WT and F204R/T243R mutant proteins. The proteins demonstrated similar spectra.

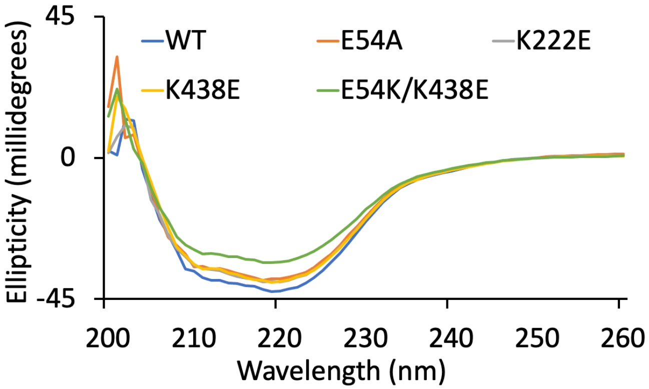

Figure 4s: Circular dichroism spectra of R699 WT, E54A, K222E, K438E, and E54K/K438E mutant proteins. The spectra were similar.

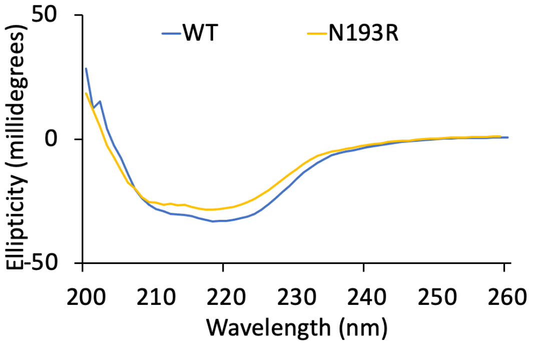

Figure 5s: Circular dichroism spectra of R699 WT and N193R mutant proteins. The proteins demonstrated similar spectra.

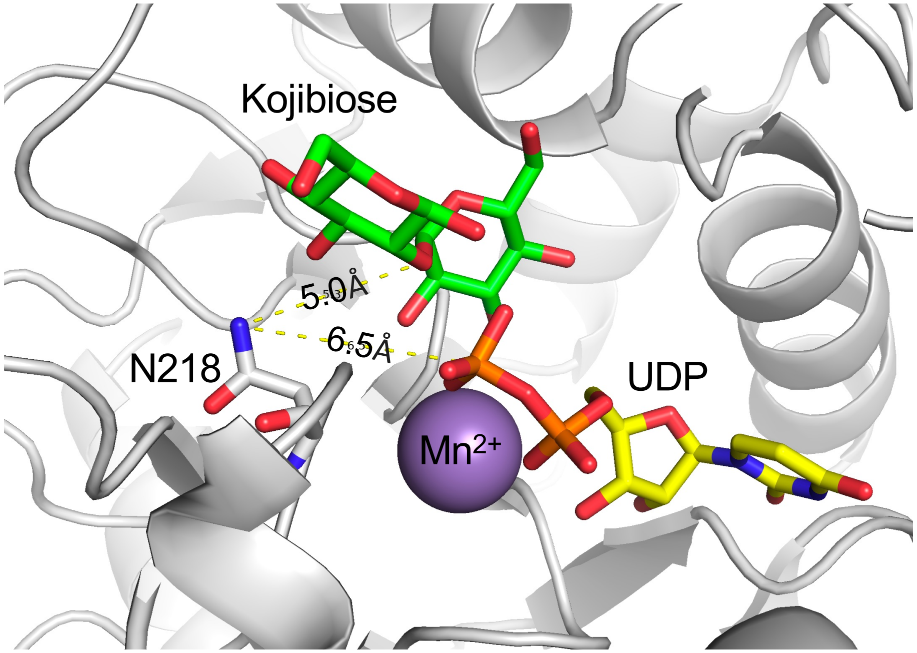

Figure 6s: R699 active site bound to Mn^{2+}, UDP, and the product kojibiose. Ribbon diagram showing distance measurement between the N218 and glycosidic linkage & UDP. Locations of Mn^{2+} (royal purple), kojibiose (green), and UDP (yellow) are indicated.

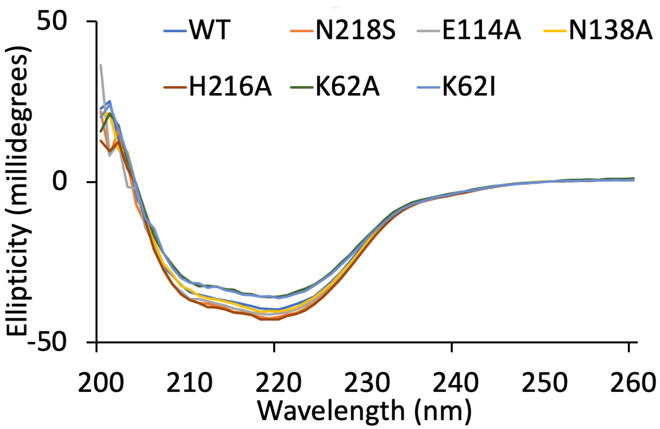

Figure 7s: Circular dichroism spectra of R699 WT, N218S, E114A, N138A, H216A, K62A, and K62I mutant proteins. The proteins demonstrated similar spectra.

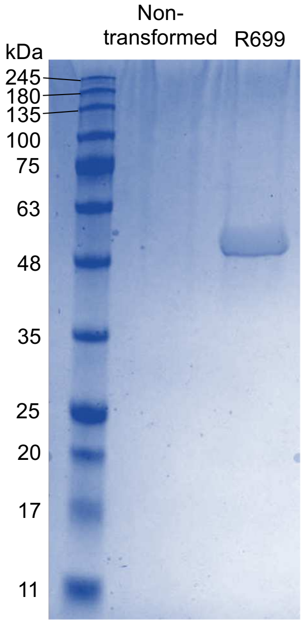

Figure 8s: SDS-polyacrylamide gel electrophoresis of the immobilized metal affinity chromatography eluates. Cell lysate samples of untransformed *E. Coli* strain MEC143 (non-transformed) and MEC143 transformed with R699 (R699) were purified using immobilized metal affinity chromatography, separated using SDS-polyacrylamide gel electrophoresis, and visualized using Coomassie blue staining. The gel was recolored in blue.

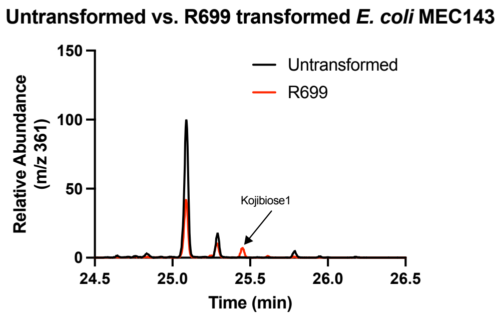

Figure 9s: Gas chromatograms of cell lysates from untransformed *E. Coli* strain MEC143 (in black) versus MEC143 transformed with R699 (in red). Gas chromatograms were generated by extracting m/z 361, a characteristic ion of methyloxime-derivatized disaccharides with eight trimethylsilyl (TMS) groups. The major peak corresponding to kojibiose is indicated. Two isomeric forms of the derivatized disaccharides are identified.

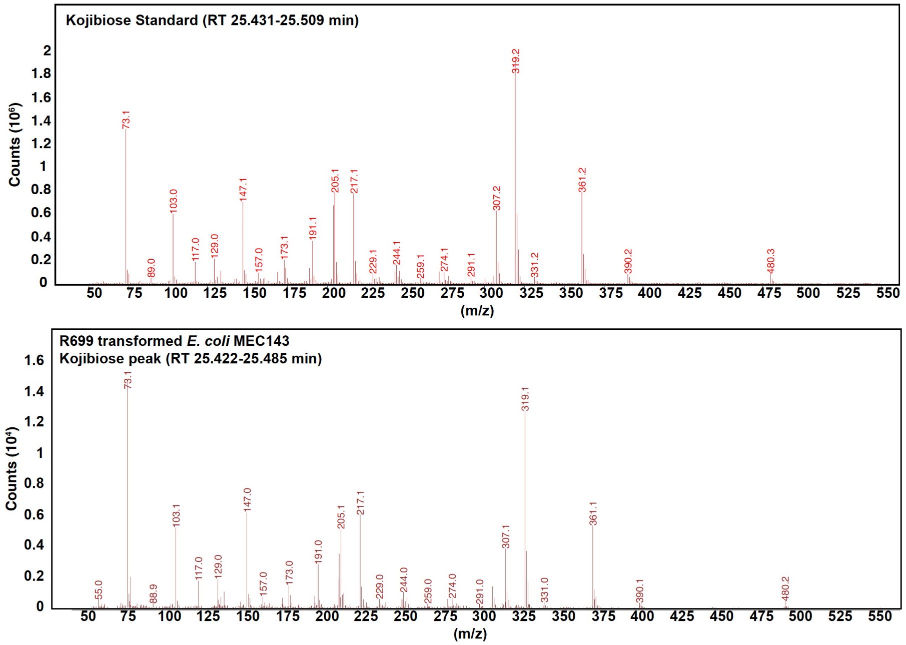

Figure 10s: MS analysis of kojibiose standard (top) and MEC143 transformed with R699 (bottom). The GC eluates of the kojibiose standard (Retention Time (RT) 25.431–25.509 min) and MEC143 transformed with R699 (RT 25.422–25.485 min) were analyzed by MS.

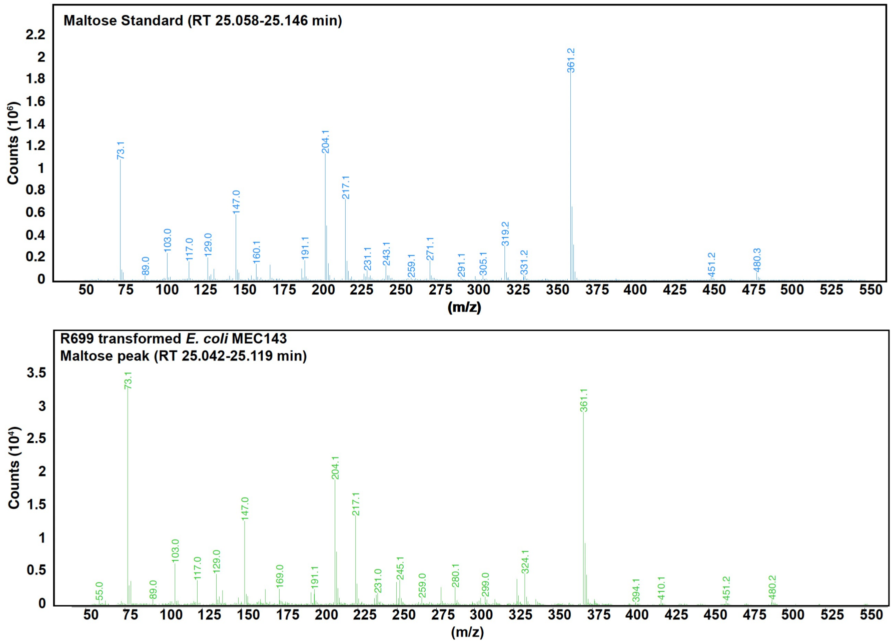

Figure 11s: MS analysis of maltose standard (top) and MEC143 transformed with R699 (bottom). The GC eluates of the maltose standard (Retention Time (RT) 25.058-25.146 min) and MEC143 transformed with R699 (RT 25.042-25.119) were detected by MS.

```
PLOD2WT    MGGCTVKPQLLLLALVLHPWNPCLGADSEKPSSIPTDKLLVITVATKESDGFHRFMQSAK     60
PLOD2B-GA  ---MGSSHHHHHHSSGLVPRGSHM-LEVLFQGPGSTDKLLVITVATKESDGFHRFMQSAK     56
PLOD2A-GA  ---MGSSHHHHHHSSGLVPRGSHM-LEVLFQGPGSTDKLLVITVATKESDGFHRFMQSAK     56
                 . :    :  * * .  :  :    .   *************************

PLOD2WT    YFNYTVKVLGQGEEWRGGDGINSIGGGQKVRLMKEVMEHYADQDDLVVMFTECFDVIFAG     120
PLOD2B-GA  YFNYTVKVLGMGEEWRGGDGINSIGGGQKVRLLKEAMEKYKDQEDLVIMFTECFDVIFAG     116
PLOD2A-GA  YFNYTVKVLGMGEEWRGGDGINSIGGGQKVRLLKEAMEKYKDQEDLVIMFTECFDVIFAG     116
           ********** *********************:**.**:* **:***:************

PLOD2WT    GPEEVLKKFQKANHKVVFAADGILWPDKRLADKYPVVHIGKRYLNSGGFIGYAPYVNRIV     180
PLOD2B-GA  GPEELLKKFQKFNHKVVFAADGILWPDKRLADKYPVVHEGKRYLNSGGFIGYAPYVYRIV     176
PLOD2A-GA  GPEELLKKFQKFNHKVVFAADGILWPDKRLADKYPVVHEGKRYLNSGGFIGYAPYVYRIV     176
           ****:****** ************************** ***************** ***

PLOD2WT    QQWNLQDNDDDQLFYTKVYIDPLKREAINITLDHKCKIFQTLNGAVDEVVLKFENGKARA     240
PLOD2B-GA  QQWNLQDNDDDQLFYTKVYIDPDKREKLNITLDHKCKIFQTLNGAVDEVVLKFENGKARA     236
PLOD2A-GA  QQWNLQDNDDDQLFYTKVYIDPDKREKLNITLDHKCKIFQTLNGAVDEVVLKFENGKARA     236
           ********************** *** :********************************

PLOD2WT    KNTFYETLPVAINGNGPTKILLNYFGNYVPNSWTQDNGCTLCEFDTVDLSAVDVHPNVSI     300
PLOD2B-GA  RNTVYDTLPVIIHGNGPTKILLNYFGNYVPNAWTQDNGCTLCEFDTVDLSAVDVYPNVLI     296
PLOD2A-GA  RNTVYDTLPVIIHGNGPTKILLNYFGNYVPNAWTQDNGCTLCEFDTVDLSAVDVYPNVLI     296
           :**.*:**** *:******************:**********************:*** *

PLOD2WT    GVFIEQPTPFLPRFLDILLTLDYPKEALKLFIHNKEVYHEKDIKVFFDKAKHEIKTIKIV     360
PLOD2B-GA  AIFIEQPTPFLPEFLDRLLTLDYPKERLKLFIHNNVEYHEKDIKKFFDKAKHEIKTIKIV     356
PLOD2A-GA  AIFIEQPTPFLPEFLDRLLTLDYPKERLKLFIHNNVEYHEKDIKKFFDKAKHEIKTIKIV     356
           .:**********.*** ********* *******:  ******* ***************

PLOD2WT    GPEENLSQAEARNMGMDFCRQDEKCDYYFSVDADVVLTNPRTLKILIEQNRKIIAPLVTR     420
PLOD2B-GA  GPEENLSEAEARNMAMDFCRQDPDCDYYFSVDADVVLTNPQTLKILIEQNRKIIAPLVTR     416
PLOD2A-GA  GPEENLSEAEARNMAMDFCRQDPDCDYYFSVDADVVLTNPQTLKILIEQNRKIIAPLVTR     416
           *******:******.******* .****************:*******************

PLOD2WT    HGKLWSNFWGALSPDGYYARSEDYVDIVQGNRVGVWNVPYMANVYLIKGKTLRSEMNERN     480
PLOD2B-GA  PGKLWSNFWGALSPDGYYARSEDYVDIVQGKRVGIWNVPYMSHVYLIKGETLRSEMNERN     476
PLOD2A-GA  PGKLWSNFWGALSPDGYYARSEDYVDIVQGKRVGIWNVPYMSHVYLIKGETLRSEMNERN     476
            *****************************:***:******::******:**********

PLOD2WT    YFVRDKLDPDMALCRNAREMTLQREKDSPTPETFQMLSPPKGVFMYISNRHEFGRLL        537
PLOD2B-GA  YFVRDKLDPDMAWCRNIREMTLQREKDSPTPETFQMLSPPKGIFMYVSNRHEFGRLL        533
PLOD2A-GA  YFVRDKLDPDMAWCRNIREM---------------------GIFMYVSNRHEFGRLL        512
           ************ *** ***                     *:***:**********
```

Figure 12s: Sequence alignment of human PLOD2b wild type (WT) and PLOD2 mutants (PLOD2a-GA and PLOD2b-GA).
